# Supplementary material for: Nonsuicidal self-injury as a mediator between dissociative experiences and suicide risk in adolescents: Insights from a clinical setting
Source: Glob Ment Health (Camb). 2025 Oct 27;12:e124. doi: 10.1017/gmh.2025.10079 (PMC12641291; doi:10.1017/gmh.2025.10079)
Supplement: Temeltürk et al. supplementary material [file S2054425125100794sup001.docx]

**APPENDIX**

**Table A1.** Descriptive results of the psychiatric scales

| **Psychiatric scales** | **Mean ± SD** |
| --- | --- |
| Adolescent Dissociative Experiences Scale |  |
| Dissociative amnesia | 28.18 ± 17.63 |
| Absorption and imaginative involvement | 26.82 ± 13.98 |
| Passive influence | 24.19 ± 12.49 |
| Depersonalization and derealization | 55.90 ± 29.94 |
| Total | 135.09 ± 67.15 |
| Inventory statements about self-injury |  |
| Otonom functions |  |
| Affect regulation | 4.17 ± 1.80 |
| Anti-suicide | 2.34 ± 1.92 |
| Marking distress | 3.04 ± 2.02 |
| Self punishment | 2.93 ± 3.13 |
| Anti dissociation | 2.29 ± 1.81 |
| Total | 14.60 ± 7.07 |
| Social functions |  |
| Intepersonal boundaries | 1.89 ± 1.69 |
| Interpersonal influence | 1.44 ± 1.54 |
| Revenge | 2.06 ± 1.63 |
| Sensation seeking | 0.98 ± 1.49 |
| Peer bounding | 0.40 ± 1.01 |
| Toughness | 1.98 ± 2.14 |
| Autonomy | 1.73 ± 2.87 |
| Self-care | 1.74 ± 1.53 |
| Total | 12.39 ± 9.53 |
| Overall total | 26.85 ± 15.11 |
| Suicide probability scale |  |
| Hopelessness | 33.76 ±7.48 |
| Suicidal ideation | 19.43 ± 6.82 |
| Negative self-evaluation | 25.46 ± 6.91 |
| Hostility | 17.42 ± 4.59 |
| Total | 96.23 ± 18.02 |
| Revised child anxiety depression scale-child version |  |
| Generalized anxiety disorder | 56.98 ± 11.95 |
| Separation anxiety disorder | 59.52 ± 12.84 |
| Panic disorder | 66.98 ± 13.39 |
| Obsessive-compulsive disorder | 62.14 ± 10.67 |
| Social phobia | 50.48 ± 14.78 |
| Major depressive disorder | 80.65 ± 16.59 |
| Total Anxiety | 60.70 ± 14.42 |
| Total Internalizing | 66.59 ± 11.46 |

SD: Standard deviation
